# High lipid storage in vacoular forms of subtype 6 *blastocystis* sp. in ostrich

**DOI:** 10.1186/s13071-014-0469-7

**Published:** 2014-10-30

**Authors:** Hemalatha Chandrasekaran, Suresh Kumar Govind, Chandrawathani Panchadcharam, Premaalatha Bathmanaban, Kalyani Raman, Gaythri Thergarajan

**Affiliations:** Department of Parasitology, Faculty of Medicine, University of Malaya, 50603 Kuala Lumpur, Malaysia; Veterinary Research Institute Ipoh, 59 Jalan Sultan Azlan Shah, 31400 Ipoh, Perak Malaysia

**Keywords:** *Blastocystis* sp, Ultrastructure, Subtype, Host susceptibility

## Abstract

**Background:**

*Blastocystis* sp., a widely prevalent intestinal protozoan parasite is found in a wide range of animals, including humans. The possibility of zoonotic transmission to human from birds especially ostriches led us to investigate on the cross infectivity of *Blastocystis* sp. isolated from the ostrich feces as well as the phenotypic and subtype characteristics. There is a need to investigate this especially with the rising number of ostrich farms due to the growing global ostrich industry.

**Findings:**

100% of the ostriches were found to be positive for *Blastocystis* sp. using the *in-vitro* cultivation method. Transmission electron microscopy revealed high electron dense material in the central body of the vacoular forms. The membrane layer of the ostrich isolate was significantly (p = 0.003) thicker as compared to human isolate. Sudan staining revealed that this was lipid accumulation. We provide evidence for the first time, the existence of subtype 6 which has been previously reported only in pigs and cattle. Cysts, ranging from 3.0 to 7.0 μm in diameter caused experimental infection in Sprague Dawley rats implicating that *Blastocystis* sp. isolated from ostriches exhibits low host specificity.

**Conclusion:**

The study for the first time demonstrates that *Blastocystis* sp. subtype 6 do exist in ostriches and show high lipid storage in the vacuoles of the parasites. The study further provides evidence for potential zoonotic transmission in ostrich farms as *Blastocystis* subtype 6 can infect rats and the same subtype have been previously reported in humans.

## Findings

The increasing number of ostrich farms led us to investigate on the possibility of cross infectivity of *Blastocystis* sp. isolated from the ostrich feces as well elucidate phenotypic and subtype characteristics. 37 fresh fecal samples of ostriches (*Struthio camelus*) and fresh human fecal sample were collected from a local ostrich farm and from an asymptomatic individual infected with *Blastocystis* sp. respectively from one of the states in Malaysia and cultured using the in-vitro culture technique using Jones’ medium and sequenced-tagged site (STS) primer-polymerase chain reaction using ten sets of primers for subtype analysis. 100% of the ostriches were found to be positive for *Blastocystis* sp. using the *in-vitro* cultivation method. Transmission electron microscopy revealed high electron dense material in the central body of the vacoular forms. We provide evidence for the first time, the existence of subtype 6 in ostriches which showed high lipid storage. Cysts, ranging from 3.0 to 7.0 μm in diameter caused experimental infection in Sprague Dawley rats implicating that *Blastocystis* sp. isolated from ostriches exhibits low host specificity. The study further provides evidence for potential zoonotic transmission in ostrich farms as *Blastocystis* subtype 6 can infect rats and the same subtype have been previously reported in humans.

## Background

*Blastocystis* sp. is a widely prevalent intestinal protozoan parasite seen in a wide of a range of animals, including humans. Previous publications have shown *Blastocystis* sp. in non-human primates, birds, chickens, ducks, geese, ostriches, amphibians, reptiles, fish, arthropods and annelids [1–6].

There are of 17 distinct subtypes (ST1-ST17) seen in humans, non-human primates, other mammals and birds [7–9]. Although only some subtypes are described to be host specific especially subtype 6 which are commonly found in pigs and cattle, however most of the subtypes are shown to exhibit low host specificity [10] which may play a role in the cross-transmission between animal and human especially humans with histories of close association to pets or farm animals [11]. Transmission is through the fecal-oral route, though waterborne, food borne and sexual transmission have been reported [12–15].

Despite a few studies showing *Blastocysis* in ostriches [16–19], there has been none that has elucidated the ultrastructural details and subtype characterization. As ostrich farming industry is increasing worldwide including Malaysia due to its economic sale of its meat, feathers, oil and leather, it is vital to ascertain of the possible potential zoonotic transmission that can be transmitted to man. It is also possible that rats can acquire the infection by traveling to human dwelling. The study attempts to further elucidate information pertaining to ultrastructural, subtype and host susceptibility of *Blastocystis* sp. isolated from domestic ostriches.

## Materials and methods

### Ethical approval

All animals used in this study were handled according to Institutional Animal Care and Use Committee (IACUC), University Malaya guidelines with the Reference. No: PAR/29/06/2012/LIL(R) and PAR/23/05/2013/HC(R). Human ethical approval for this study was obtained in accordance with University Malaya Medical Centre research policy with Reference. No: 926.7.

### Animals and management

A local ostrich farm from one of the states in Malaysia was selected for the study. The farm practiced intensive type of management where the animals are confined in the same pen. Ostriches were kept in pairs or three’s for breeding purpose in well-fenced housing pens. The animals were fed with quail layer mash, napier grass and given ad libitum water.

### Source of *Blastocystis* sp. isolates

A total of thirthy-seven (n = 37) fresh fecal samples of ostriches (*Struthio camelus*) and fresh human fecal sample were collected from a local ostrich farm and from an asymptomatic individual infected with *Blastocystis* sp. respectively from one of the states in Malaysia. The samples were collected in stool collection container and were processed as soon after collection.

### Laboratory testing

#### *In vitro cultivation of Blastocystis* sp. *isolates*

The parasites were isolated from the fecal samples of ostriches and human by *in-vitro* cultivation using Jones’ medium supplemented with 10% heat-inactivated horse serum at 37°C. Subsequently after isolation, the parasites were maintained in Jones’ medium by consecutive sub-cultures every 3 to 4 days for at least one month prior to phenotypic, subtype and ultrastructural analysis [6,20].

#### Floatation method

Approximately one gram of fresh feces, emulsified with saturated salt solution was then filtered through gauze into a centrifuge tube. Saturated salt solution was then later added up to the meniscus of test tube before lowering a coverslip onto the top of the tube. Coverslip was then lifted vertically up and placed onto a clean slide. Samples were observed under 10x objective lens of compound microscope for the presence of helminth ova, nematode eggs, coccidia oocysts and other parasites [21].

#### Transmission electron microscopy

Parasites isolated from ostriches and human were chosen for the ultrastructural studies. The contents of day 3 culture were washed three times using phosphate buffered saline (PBS) pH 7.4. The samples were centrifuged at 3000rpm for 5 minutes. The pelleted cells were re-suspended overnight in 2.5% glutaraldehyde in 0.1 M sodium cacodylate buffer, pH 7.3 at 4°C, washed thoroughly with cacodylate buffer and post fixed for 30 min in 1% osmium tetroxide in cacodylate buffer. The fixed cells were dehydrated for 5 minutes in ascending series of ethanols (30%, 50%, 70%, 80%, 90% and 100%) and embedded in epoxy resin. Semi-thin sections were stained with toluidine blue. Ultrathin sections were cut, contrasted with uranyl acetate and lead citrate and viewed using a transmission electron microscope (LEO Libra120) [22].

#### Sudan Black B staining

Parasites from day 3 culture of the ostrich and human isolates were smeared on a clean glass slide and immediately dried with a hair dryer at room temperature. Then the cells were fixed with 4% glutaraldehyde fixative solution in borate buffer, pH 7.6 for 1 minute at 2-8°C with gentle agitation followed by thorough rinsing in deionized water. The cells were then stained with Sudan Black B reagent by immersing slides for 5 minutes with intermittent agitation. The cells were then rinsed 3 to 5 times in 70% ethanol followed by a thorough rinsing in distilled water. After rinsing, the cells were counterstained in haematoxylin solution for 5 minutes followed by thorough rinsing in tap water. Slides were then examined under a conventional Olympus microscope equipped with an immersion oil objective lens (100x) for the presence of black droplets in the central vacuole indicating positive reactions. The samples were stained using a commercial solution, Sudan Black B Staining System (Sigma Aldrich, Germany) according to the recommendations of the manufacturer.

### Molecular detection

#### Genomic DNA preparation

DNA was extracted from the culture sample of all 37 ostrich isolates and a human isolate using QIAamp DNA Stool Mini Kit (QIAGEN, Hilden, Germany) according to the manufacturer’s protocol [4].

#### Subtyping of Blastocystis sp. isolates

All 37 parasite isolates were subjected to sequenced-tagged site (STS) primer-polymerase chain reaction using the following ten sets of primers [4] (Table 1). Two to five μl of DNA preparations were used to amplify the genomic sequences in a 20 μl reaction containing 0.5 mM of the dNTPs, 0.5 mM of each primer, 1 × PCR buffer (75 mM Tris-HCL, pH 8.8, 20 mM (NH_4_)_2_SO_4_ and 0.01% Tween 20), 2.5 mMm MgCl_2_ and 1 U Taq DNA Polymerase (recombinant) (FERMENTAS, USA). PCR conditions consisted of 1 cycle of initial denaturing at 94°C for 3 minutes, followed by 30 cycles including denaturing at 94°C for 30 s, annealing at 57°C for 30 s and extending at 72°C for 1 minute, and an additional cycle with a 10 min chain elongation at 72°C (thermocycler Eppendorf, Germany). The amplification products were electrophoresed in 1.5% agarose gels (PROMEGA USA) and Tris-Borate-EDTA buffer. Gels were stained with ethadium bromide and photographed using ultra-violet gel documentation system (Uvitec, United Kingdom). The PCR amplication for each primer pair was repeated thrice for each isolate [4]. The classification of the subtypes for each *Blastocystis* sp. isolate was based on the standard terminology [8].

**Table 1:** **List of sequenced-tagged site (STS) primers**

**Subtype**	**STS primer**	**Product size (bp)**	**Sequence of forward (F) and reverse (R) primer (5’ – 3’)**	**Genebank accession no.**
1	SB82	462	F-TCTTGCTTCATCGGAGTC	AF166085
			R-CCTTCTCGCAGTTCTTTATC	
1	SB83	351	F-GAAGGACTCTCTGACGATGA	AF166086
			R-GTCCAAATGAAAGGCAGC	
2	SB155	650	F-ATCAGCCTACAATCTCCTC	AF166087
			R-ATCGCCACTTCTCCAAT	
3	SB227	526	F-TAGGATTTGGTGTTTGGAGA	AF166088
			R-TTAGAAGTGAAGGAGATGGAAG	
3	SB228	473	F-GACTCCAGAAACTCGCAGAC	AF166089
			R-TCTTGTTTCCCCAGTTATCC	
3	SB229	631	F-CACTGTGTCGTCATTGTTTTG	AF166090
			R-AGGGCTGCATAATAGAGTGG	
4	SB332	338	F-GCATCCAGACTACTATCAACATT	AF166091
			R-CCATTTTCAGACAACCACTTA	
5	SB340	704	F-TGTTCTTGTGTCTTCTCAGCTC	AY048752
			R-TTCTTTCACACTCCCGTCAT	
6	SB336	317	F-GTGGGTAGAGGAAGGAAAACA	AY048751
			R-GAACAAGTCGATGAAGTGAGAT	
7	SB337	487	F-GTCTTTCCCTGTCTATTCTGCA	AY048750
			R-AATTCGGTCTGCTTCTTCTG	

### *In vivo* study

The cysts, isolated from fresh fecal material of two respective ostriches, collected in separate tubes by using Ficoll Paque method were made to a concentration of 10^5^ of cysts/ml. This was then inoculated orally, using 20G feeding needle of 1.5inch length into nine Sprague Dawley rats. All the 9 rats were divided into two respective groups, Group A of 5 rats and Group B of 4 rats and were anesthetized before inoculation of respective strains. Stools were collected from the rats to examine for the presence of *Blastocystis* sp., two days of post-inoculation [23]. Samples found positive for *Blastocystis* sp. were then subjected to subtyping.

### Statistical analysis

Statistical analysis was carried out using IBM^©^ SPSS^©^ Statistics Version 21. Independent Students t-test was used to assess the differences in the membrane thickness of *Blastocystis* sp. isolated from ostrich and human. A value of p < 0.05 is considered statistically significant.

## Results

### Prevalence

100% of 37 ostriches were found to be positive for *Blastocystis* sp. No other parasites were seen. However all ostriches appeared healthy without any symptoms. Direct microscopy of fecal smears revealed 8-12 *Blastocystis* sp. under 40x magnification (Table 2).

**Table 2:** **Prevalence of** ***Blastocystis*** **sp. in ostrich isolates**

**Sex of host**	**Number of animals**	**Number of animals detected positive**
**Male**	20	20
**Female**	17	17

### Ultrastructural studies of *Blastocystis* sp.

**Figure 1:**
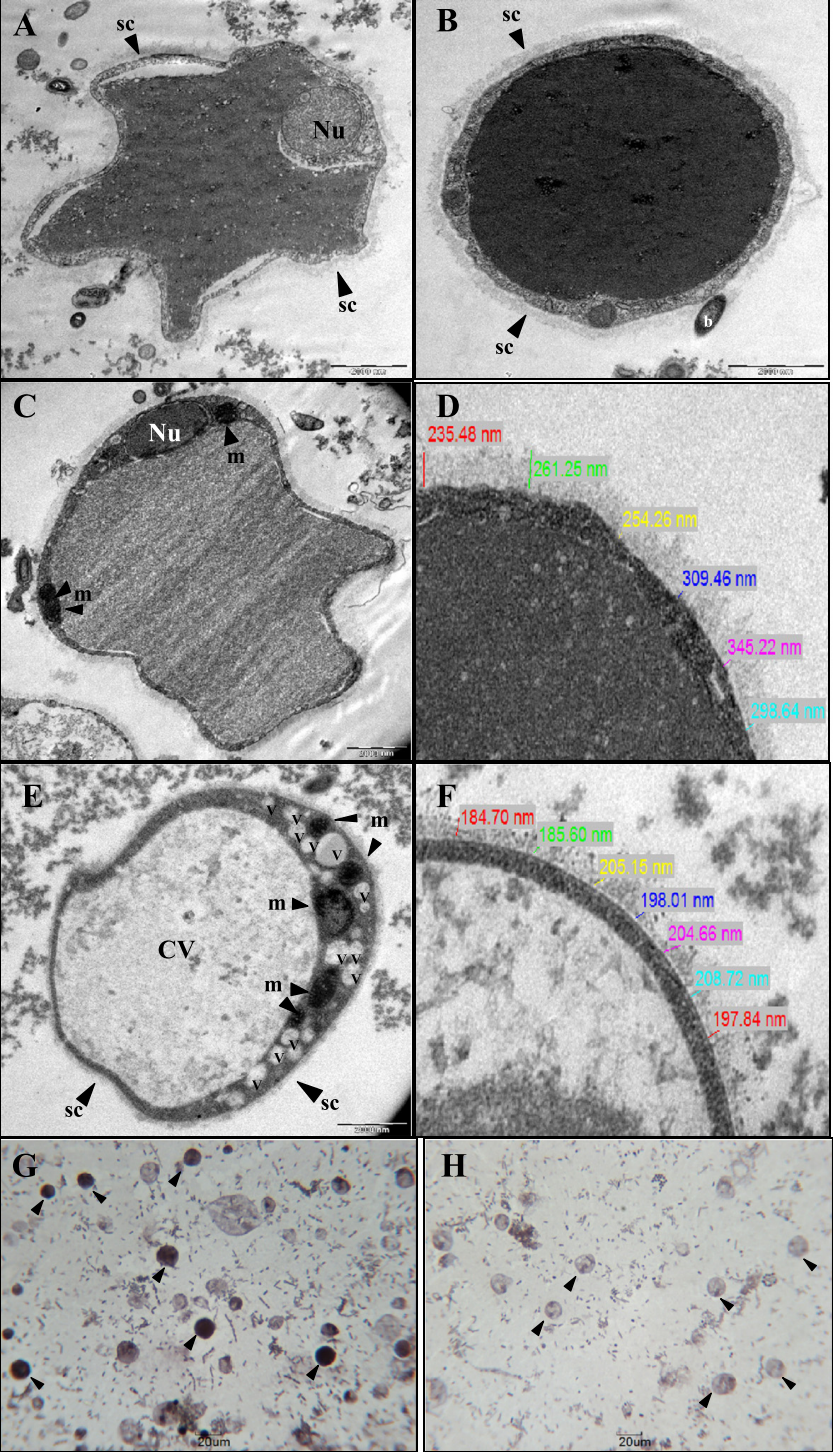
**Comparison of Transmission electron microscopy and light microscopy of** ***Blastocystis*** **sp. for ostrich isolates and human isolates. A**: Transmission electron micrograph showing an irregular shape *Blastocystis* sp. with a prominent nucleus (*Nu*) **B**: A thick, compact surface coat (*sc*) is seen to surround the cell when examined by transmission electron microscopy. A high electron dense area was observed in the central vacuole (*CV*). **C**: Numerous mitochondria (*m*) were seen in the *Blastocystis* sp*.* cells of the ostrich isolates. **D**: Higher magnification of *Blastocystis* sp. membrane in the ostrich faecal culture. **E**: A multi-vacoulated form (v) of *Blastocystis* sp. in human faecal culture with multiple mitochondria present in the cytoplasm. **F**: Higher magnification of *Blastocystis* sp. membrane in the human faecal culture. Note: the cell membrane of *Blastocystis* sp. in ostrich and human isolates were 235.48 to 345.22 nm and 184.70 to 208.72 nm, respectively. **G**: Light microscopic images of *Blastocystis* sp. isolated on day 3 of ostrich faecal culture stained with Sudan Black B. Positive reactions are seen as dark droplets in the central vacuole. Note: dark droplets (*arrows*). **H**: Light microscopic images of *Blastocystis* sp. isolated on day 3 of human faecal culture stained with Sudan Black B. No reactions were observed in the central vacuole.

Transmission electron micrographs of *Blastocystis* sp. showed slight irregular in shape with a thick and compact surface coat seen to be surrounding the cell (Figure 1A). Bacteria were occasionally seen to be adhering to the surface coat (Figure 1B). High electron dense material was observed in the central vacuole with the presence of two prominent nuclei in most of the parasites (Figure 1B). Numerous mitochondria were seen in the *Blastocystis* sp. cells of the ostrich isolates (Figure 1C). Meanwhile, *Blastocystis* sp. in human fecal culture (Figure 1E) illustrates a multi-vacoulated form of this organism with multiple mitochondria present in the cytoplasm and a clear, large central vacuole (CV). The membrane layer of the ostrich isolate (Figure 1D) was significantly (p = 0.003) thicker, 284.05 ± 40.91 nm (range, 235.48 to 345.22 nm) as compared to human isolate (Figure 1F), 197.81 ± 9.49 nm (range, 184.70 to 208.72 nm) (Table 3). Parasites from day 3 cultures showed ostrich isolate stained with Sudan Black B, positive reactions were observed in the central vacuole of *Blastocystis* sp. (Figure 1G) meanwhile no reactions were observed in the central vacuole of the *Blastocystis* sp. from the day 3 human isolate (Figure 1H). Positive reactions are seen as dark droplets in the central vacoule (CV).

**Table 3:** **Statistical comparison of membrane thickness of** ***Blastocystis*** **sp. isolated from ostrich and human (p < 0.05)**

	**Organism**	**Range**	**Means ± S.D**	**p-value**
**Thickness of membrane (nm)**	Ostrich	235.48 - 345.22	284.05 ± 40.91	0.003
	Human	184.70 - 208.72	197.81 ± 9.49	

### Subtyping of *Blastocystis* sp.

**Figure 2:**
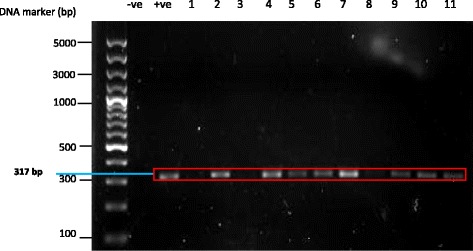
**Examples of polymerase chain reaction (PCR) products from isolates of** ***Blastocystis*** **sp** ***.*** **amplified by sequenced-tagged site (STS) primers.** Lane 1, DNA size markers of a 100-bp DNA ladder plus; Lane 2, negative control; Lane 3, positive control; Lane 5, Lanes 7-10, Lanes 12-14; subtype 6 (317 bp).

Using sequenced-tagged site primer-PCR, 14 of the ostrich isolates were confirmed to be subtype 6 (Figure 2). Meanwhile, the subtype of remaining 23 isolates when amplified with PCR using sequence-tagged site (STS) revealed no bands when assessed using primers for subtypes 1 to 7.

### In vivo study

**Figure 3:**
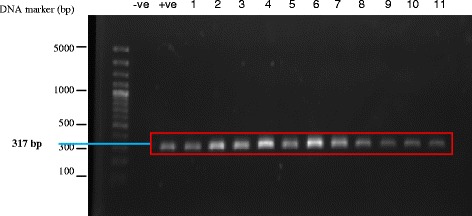
**Examples of polymerase chain reaction (PCR) products from isolates of** ***Blastocystis sp.*** **from the cross-infection** ***in vivo*** **study amplified by sequenced-tagged site (STS) primers.** Lane 1, DNA size markers of a 100-bp DNA ladder plus; Lane 2, negative control; Lane 3, positive control; Lanes 4-5, *Blastocystis* sp. cyst of ostrich isolate (inoculum); Lane 6-14, *Blastocystis* sp. isolates of infected rats; subtype 6 (317 bp).

The *Blastocystis* cysts isolated from fresh fecal samples of ostriches ranged from 3.0 to 7.0 μm in diameter. These cysts caused experimental infection in Sprague Dawley rats. *Blastocystis* sp. was detected in feces of experimentally infected Sprague Dawley rats, two days of post-inoculation. The rats infected with ostrich isolate were identified to be subtype 6 which was similar to the strain of the inoculum when amplified with PCR using sequence-tagged site (STS) primers (Figure 3).

## Discussion

A high prevalence of Blastocystis infection with 100% positive in the ostrich population indicates that this organism is commonly found in this avian. Ultrastructural studies concurred with the previous studies [24,25]. Most of the cells appeared to be rounded or slightly irregular in shape with a thick and compact surface coat seen to be surrounding the cell. One or more nuclei with numerous mitochondria were commonly observed in the cytoplasm of the organism [5]. The outer membrane of ostrich isolate was observed to be significantly thicker when compared to human isolate possibly conferring greater resistance in non-conducive environments. The most distinguishing characteristic was the presence of high electron dense material within the vacuolar forms of parasites on day 3 cultures. Sudan Black B revealed in more than 50% of the parasites dark stains in portions of the central vacuole indicating the presence of neutral lipid [26]. The study confirms that *Blastocystis* sp. from ostrich isolates uses the vacuolar forms to store lipids.

Although subtype 6 was seen in livestock animals [9] especially in pigs and cattle [27] this is the first study to demonstrate subtype 6 seen in ostrich isolates when amplified with polymerase chain reaction using sequence-tagged site (STS) primers (Figure 2). Roberts *et al*. [19] showed that 6 out of 10 ostriches examined for *Blastocystis* sp. were subtype 7. In the present study, the *Blastocystis* cysts of ostrich isolate were able to cause experimental infection in Sprague Dawley rats (Figure 3) as evidenced by the subtype 6 seen in the stools of infected rats which were prior negative for *Blastocystis* sp. Subtype 6 have been shown to be seen in humans [28] and therefore can be postulated that the ostrich farms with wild rats can be a reservoir for human infections since *Blastocystis* sp. exhibits low host specificity.

## Conclusion

The present study is the first to elucidate the ultrastructural, subtype, and host susceptibility of *Blastocystis* sp. isolated from ostriches in order to determine the true pathogenicity of this zoonotic parasite which is known to be a potential source for cross-transmission between animal and humans especially when in close association.
